# Bone Sclerostin and Dickkopf-related protein-1 are positively correlated with bone mineral density, bone microarchitecture, and bone strength in postmenopausal osteoporosis

**DOI:** 10.1186/s12891-021-04365-8

**Published:** 2021-05-25

**Authors:** Jia Peng, Zhang Dong, Zhang Hui, Wang Aifei, Deng Lianfu, Xu Youjia

**Affiliations:** 1 Orthopedic Department, Second Affiliated Hospital of Soochow University, Suzhou, China; 2 Second Affiliated Hospital of Soochow University, Osteoporosis Research Institute of Soochow University, Suzhou, China; 3 Shanghai Institute of Traumatology and Orthopedics, Shanghai, China; 4 Shanghai Key Laboratory for Prevention and Treatment of Bone and Joint Diseases with Integrated Chinese-Western Medicine, Shanghai, China; 5 Ruijin Hospital, Jiao Tong University School of Medicine, Shanghai, China

**Keywords:** Sclerostin, Dickkopf-related protein-1, Postmenopausal osteoporosis, Bone mineral density, Bone microarchitecture, Bone strength

## Abstract

**Background:**

Wnt-catenin signaling antagonists sclerostin and dickkopf-related protein-1 (Dkk-1) inhibit bone formation and are involved in the pathogenesis of postmenopausal osteoporosis (PO). However, the association between sclerostin and Dkk-1 and bone mineral density (BMD) in women with PO remains unclear.

**Objective:**

This study aimed to determine the association between sclerostin and Dkk-1 and BMD, bone microarchitecture, and bone strength in PO.

**Methods:**

Trabecular bone specimens were obtained from the femoral heads of 76 Chinese women with PO who underwent hip arthroplasty for femoral neck fractures. Micro-computed tomography (Micro-CT) was used to assess the BMD and bone microarchitecture of the trabecular bone. Subsequently, a mechanical test was performed. Finally, sclerostin and Dkk-1 in the bone were measured by enzyme-linked immunosorbent (Elisa) assay. Serum ionized serum ionised calcium, propeptide of type 1 collagen, C-terminal β-telopeptide of type-1 collagen, sclerostin, and Dkk-1 were also detected.

**Results:**

Bone sclerostin was positively correlated with serum ionised calcium, serum sclerostin, BMD, bone volume/tissue volume (BV/TV), trabecular number (Tb.N), maximum compressive force, and yield strength (*r* = 0.32, 0.906, 0.355, 0.401, 0.329, 0.355, and 0.293, respectively, *P* < 0.05) and negatively correlated with age and trabecular separation (Tb.Sp) (*r* = − 0.755 and − 0.503, respectively, *P* < 0.05). Bone Dkk-1 was positively correlated with serum ionised calcium, serum Dkk-1, BMD, BV/TV, trabecular thickness, Tb.N, maximum compressive force, yield strength, and Young’s modulus (*r* = 0.38, 0.809, 0.293, 0.293, 0.228, 0.318, 0.352, 0.315, and 0.266, respectively, *P* < 0.05) and negatively correlated with age and Tb.Sp (*r* = − 0.56 and − 0.38, respectively, *P* < 0.05). Serum levels of sclerostin and Dkk-1 reflected the levels of sclerostin and Dkk-1 in the bone.

**Conclusion:**

Bone sclerostin and Dkk-1 were positively correlated with BMD in women with PO, and higher levels of bone sclerostin and Dkk-1 might predict better BMD, bone microarchitecture, and bone strength. The potential molecular mechanisms still require further study.

## Background

Osteoporosis is a major bone metabolic disease characterized by low bone mass and deterioration of the bone microarchitecture, resulting in fragility and increased risk of fracture. Postmenopausal osteoporosis (PO), the most common type of osteoporosis, is mainly caused by imbalanced bone turnover, which is characterized by enhanced bone resorption exceeding bone formation following estrogen decline [1]. A large number of studies have reported the critical roles of osteoblasts and osteoclasts, which are the main executors of bone formation and bone resorption, respectively, in the pathogenesis of PO. Recent breakthrough studies highlighted the central role of osteocytes in bone remodeling and revealed that osteoblasts and osteoclasts are under the control of osteocytes [2, 3].

The canonical Wnt pathway plays a key role in determining the fate of mesenchymal stem cells, which favors the maturation and survival of osteoblasts [4]. In addition, the Wnt pathway promotes the expression of osteoprotegerin rather than RANKL, thus indirectly inhibiting osteoclast activity [5, 6]. The canonical Wnt pathway in the bone is mainly regulated by osteocytes, which act through the production of sclerostin and Dickkopf-related protein 1 (Dkk-1), [7–10]. By preventing the binding between Wnt and specific cell surface receptors (Frizzled and lipoprotein receptor-related protein 5/6), sclerostin and Dkk-1 block the activation of the Wnt canonical pathway; furthermore, β-catenin is sequestered and degraded in the absence of Wnt. Therefore, sclerostin and Dkk-1 are potent inhibitors of bone formation [4, 11, 12].

In vitro evidence has shown that estrogen inhibits the expression of sclerostin in osteocytes, mesenchymal stromal cells, and osteoblastic cells [13–15]. In addition, in vivo data from an ovariectomized mouse model and clinical population investigations indicated that sclerostin levels are negatively correlated with estrogen levels [16–18]. Conversely, sclerostin antibody has been shown to significantly increase bone mineral density (BMD) in women with PO [19–21]. Similarly, Dkk-1 antibody has been demonstrated to aid the recovery of bone formation in animal models [22]. In conclusion, sclerostin and Dkk-1 play pivotal roles in the pathogenesis of PO and have emerged as valuable treatment targets.

However, the association between sclerostin and Dkk-1 and BMD in postmenopausal women remains controversial. Reportedly, as blood sclerostin levels increase, BMD decreases in postmenopausal women, and postmenopausal women with significantly increased serum Dkk-1 exhibit more significant osteoporosis [23, 24]. Other studies have suggested paradoxical associations between circulating sclerostin and Dkk-1 and BMD, revealing that serum sclerostin and Dkk-1 are positively associated with BMD in women with PO [25–29].

Although sclerostin is mainly secreted by osteocytes, recent studies have demonstrated that other cells and tissues, including osteoblasts, vascular smooth muscle cells, and livers, are also sources of sclerostin [30, 31]; therefore, we speculated that the serum levels of sclerostin and Dkk-1 could not reflect those in the bone. Because sclerostin and Dkk-1 levels reported in most previous studies were measured in the serum, we measured sclerostin and Dkk-1 levels directly in the bone tissue and further elucidated the associations between bone sclerostin and Dkk-1 and BMD, bone microarchitecture, and bone strength.

## Methods

### Subjects

From January 1 to December 31, 2018, 76 postmenopausal women who underwent hip arthroplasty for femoral neck fracture were recruited for the present study (mean age, 74.66 ± 9.23 years; range, 56–92 years). All patients suffered from fracture due to falling (fragility fracture) and not violent trauma. Exclusion criteria were as follows: (1) presence of known metabolic or bone disorders that could affect bone metabolism and BMD, such as diabetes, parathyroid disease, and severe renal impairment; (2) presence of known joint diseases that could influence bone architecture and quality, such as osteoarthritis, rheumatoid arthritis, and congenital hip dysplasia; (3) receiving drugs that affect bone metabolism, such as hormones, steroids, diphosphate and calcitonin; and (4) hip surgery history.

### Specimen preparation

The trabecular bone was obtained according to a previously reported method [32, 33]. Briefly, subchondral bone columns (15 mm in diameter and 30 mm in height) were extracted using a coring reamer at a position 15 mm outside the lateral edge of the fovea of the capitis femoris, and the axes of the specimens were perpendicular to the articular surface. The specimens were wrapped in gauze containing normal saline and stored at − 80 °C, which were prepared for mechanical testing and micro-CT scanning. Bone samples (0.05 g) from each specimen were obtained from the remaining trabecular bone in the femoral head and stored in protein lysate containing a protease inhibitor to prevent protein degradation.

### Imaging

The specimens were positioned with gauze in the sample holder and allowed to reach room temperature. Densitometric and morphometric micro-CT analyses were performed after calibrated by SP-4004(Shysan Bruker, Belgium). The bone was analyzed using a Skyscan 1172 (Skyscan, Bruker, Belgium) with a 9-μm voxel size, 59 KVp, 127 uA, and 0.48 rotation steps. Cone-beam reconstruction software version 2.6, based on the Feldkamp algorithm, was used for three-dimensional reconstruction and data processing. The trabecular region of interest was 10 mm in diameter and 30 mm in height (to exclude the external compression part of the trabecular columns). The following parameters of the specimens were calculated: BMD (g/cm^3^), bone volume/tissue volume (BV/TV, %), trabecular thickness (Tb.Th, mm), trabecular number (Tb.N, 1/mm), and trabecular separation (Tb.Sp, mm).

### Mechanical testing

A vertical compression test was performed to assess the mechanical properties of the trabecular bone after the micro-CT scan following after calibrated standard product from Sawbone. Briefly, the bone core was compressed in the inferosuperior direction between two platens at 2 mm/min on an Instron 5569 materials testing machine (Instron Inc., Norwood, MA, USA). Three parameters, including the maximum compression force (Mac, N), compressive strength (Cos, MPa), and Young’s modulus (Yom, MPa), were recorded.

### Determination of sclerostin and Dkk-1 levels in the bone

Bone samples (0.05 g) were ground in liquid nitrogen. The bone powder was then lysed in a 1.5 mL tube (the volume of protein lysate in each tube was 1 mL) on a shaking table overnight at 4 °C. The next morning, protein lysate containing a protease inhibitor was added to each tube to ensure that the total volume of the solution in the tube was 1.5 mL. Sclerostin and Dkk-1 levels were measured by enzyme-linked immunosorbent assay (ELISA; R&D Systems, Minneapolis, MN, USA), following the manufacturer’s protocols. The sclerostin and Dkk-1 levels in the trabecular bone were calculated and expressed in μg/g (X μg sclerostin/X g trabecular bone and X ug Dkk-1/ X g trabecular bone).

### Serum biochemistry

Serum was collected in the next morning after admission before the patients had breakfast. The markers were measured at the clinical laboratory of the Second Affiliated Hospital of Soochow University. Propeptide of type 1 collagen (P1NP) and C-terminal β-telopeptide of type-1 collagen (β-CTX) were measured using IDS-iSYS assays (Immunodiagnosis System Ltd., East Boldon, UK). Serum sclerostin (sensitivity:3.8 pg/L, specificity:natural and recombinant human SOST) and Dkk-1(sensitivity:0.948 pg/ml, specificity:natural and recombinant human Dkk-1) levels were measured with ELISA (R&D Systems), following the manufacturer’s protocols.

### Statistical analysis

Statistical analysis was performed using SPSS ver.16.0 (SPSS, Chicago, IL, USA). The correlations among age, bone turnover markers, bone sclerostin and Dkk-1, bone microarchitecture, and bone strength were analyzed using Spearman’s rank correlation coefficient test. The level of statistical significance was established at *P* < 0.05 for all analyses.

## Results

### Descriptive statistical results

Descriptive statistical results are listed in Table 1. The levels of sclerostin and Dkk-1 in the trabecular bone of the femoral head of Chinese postmenopausal women were 57.54 ± 11.32 μg/g and 1.15 ± 0.38 μg/g, respectively.

**Table 1:** Description statistical results

	Median (25th,75th percentile)
Age(y)	74.5 (68.00, 81.75)
calcium ion (uM)	2.08 (1.99, 2.19)
P1NP(ug/L)	47.2 (2.08, 2.20)
CTX (ug/L)	516. 481.1 (281.25, 694.73)
Serum sclerostin (ug/L)	1.02 (0.68, 1.64)
Serum DKK (ug/L)	0.02 (0.16, 0.26)
Bone sclerostin (ug/g)	58.53 (48.70, 67.00)
Bone DKK-1(ug/g)	1.12 (0.95, 1.34)
BMD (g/cm^3^)	0.64 (0.60, 0.68)
BV/TV (%)	52.07 (48.04, 56.93)
Tb.Th (mm)	0.30 (0.28, 0.32)
TB.N(1/mm)	1.10 (0.97, 1.20)
Tb.SP (mm)	0.60 (0.54, 0.69)
Mac(N)	62.44 (59.45, 68.12)
Cos (Mpa)	3.24 (2.99, 3.54)
Yom (Mpa)	29,837 (27,893, 31,284)

### Association of serum sclerostin and Dkk-1 levels and bone sclerostin and Dkk-1 levels

**Fig. 1:**
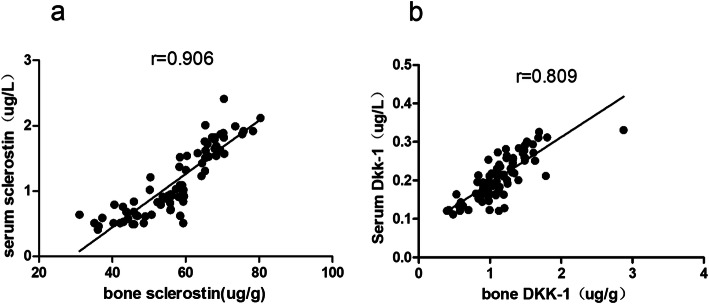
**a** The serum levels of sclerostin was positively correlated with bone sclerostin levels (*P* < 0.05). **b** The serum levels of Dkk-1 were positively correlated with bone Dkk-1 levels (*P* < 0.05)

The serum level of sclerostin was positively correlated with bone sclerostin level (*r* = 0.906, *P =* 0.000, Fig. 1a), and serum levels of Dkk-1 were positively correlated with bone Dkk-1 level (*r* = 0.809, *P =* 0.000, Fig. 1b).

### Correlation of bone sclerostin and Dkk-1 with age, serum ionised calcium, P1NP, and β-CTX

**Fig. 2:**
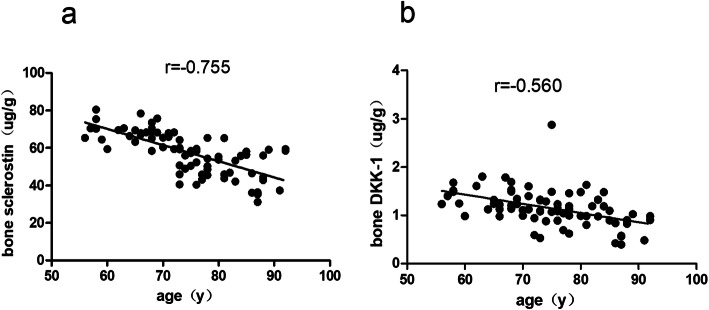
**a**-**b** Age was negatively correlated with bone sclerostin and bone Dkk-1 (*P* < 0.05)

As shown in Table 2, age was negatively correlated with bone sclerostin (*r* = − 0.755, *P =* 0.000, Fig. 2a) and Dkk-1 (*r* = − 0.560, *P =* 0.000, Fig. 2b) levels. Serum ionised calciums were positively correlated with bone sclerostin (*r* = 0.320, *P =* 0.005) and Dkk-1 (*r* = 0.293, *P =* 0.01) levels. Although there was no significant association between P1NP and β-CTX and bone sclerostin and Dkk-1 (*P > 0.05*), bone Dkk-1 seemed to be negatively correlated with P1NP (*P* = 0.054).

**Table 2:** Correlation of bone sclerostin and Dkk-1 between age, calcium ion, P1NP and CTX

	Sclerostin (ug/g)		Dkk-1(ug/g)	
	r	*P*	r	*P*
Age(y)	−0.755	< 0.05	−0.560	< 0.05
Calcium ion (uM)	0.320	< 0.05	0.293	< 0.05
P1NP(ug/L)	0.028	> 0.05	−0.222	> 0.05
CTX (ug/L)	0.070	> 0.05	−0.074	> 0.05

### Correlation of bone sclerostin and Dkk-1 with BMD, bone microarchitecture, and bone strength

**Fig. 3:**
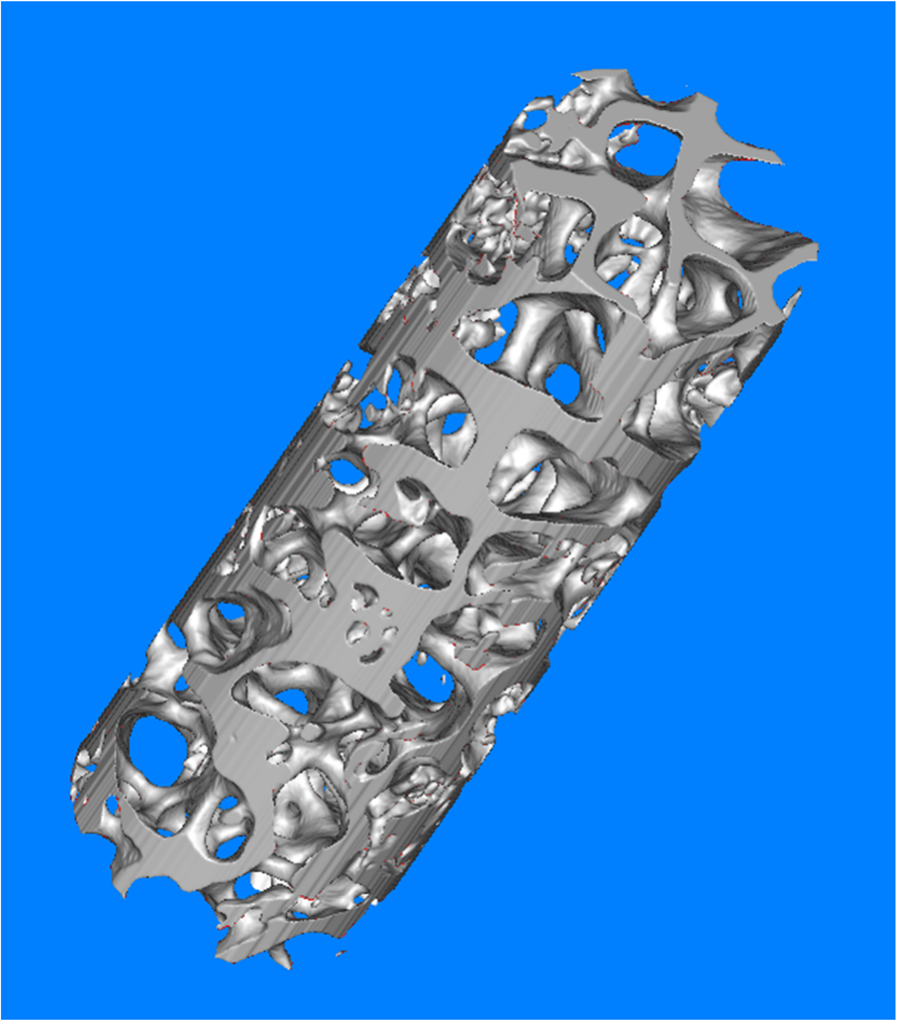
The representative Micro-CT picture of trabecular bone from femoral head

**Fig. 4:**
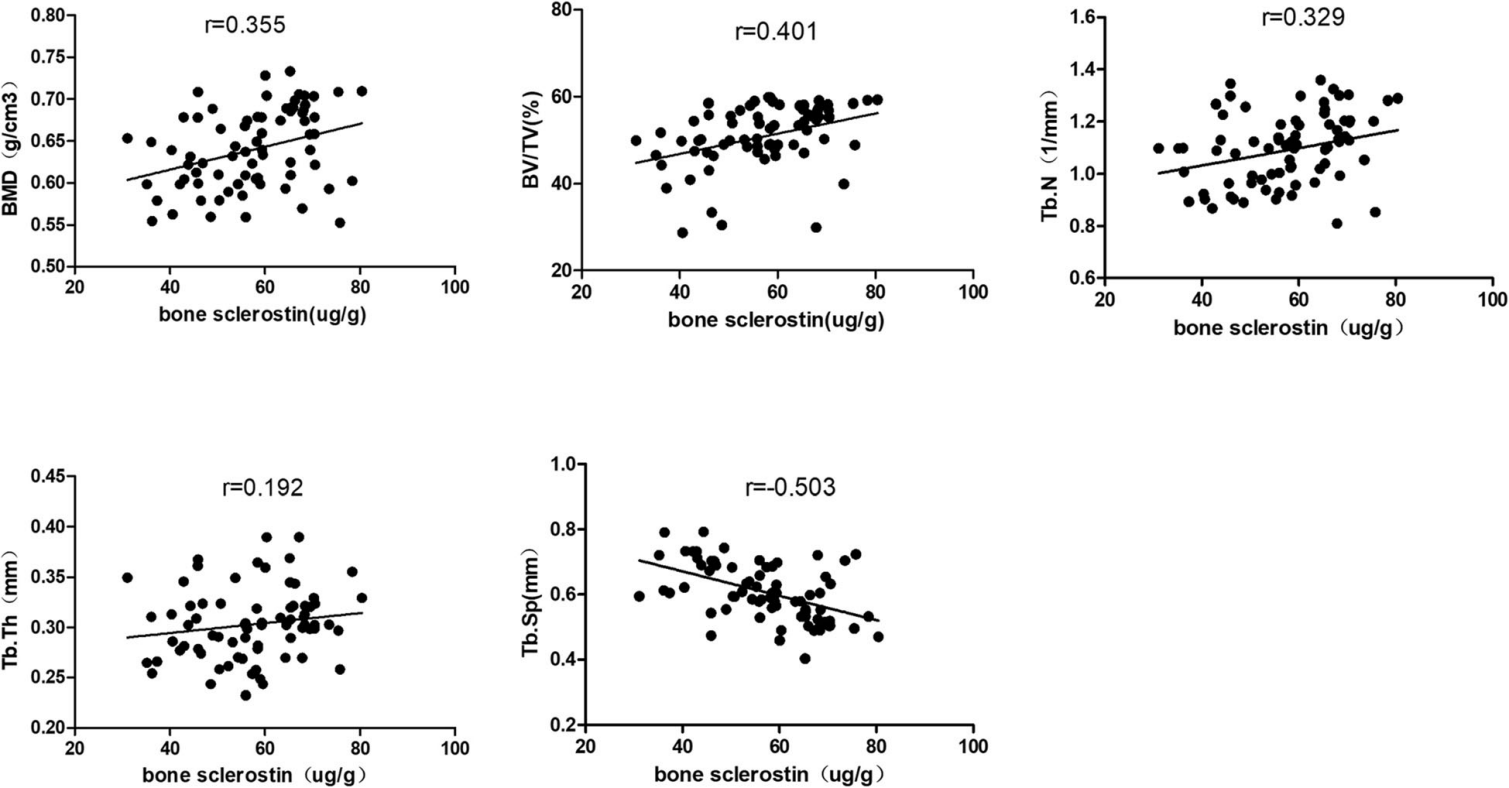
The correlationship of bone sclerostin and BMD, bone microarchitecture

**Fig. 5:**
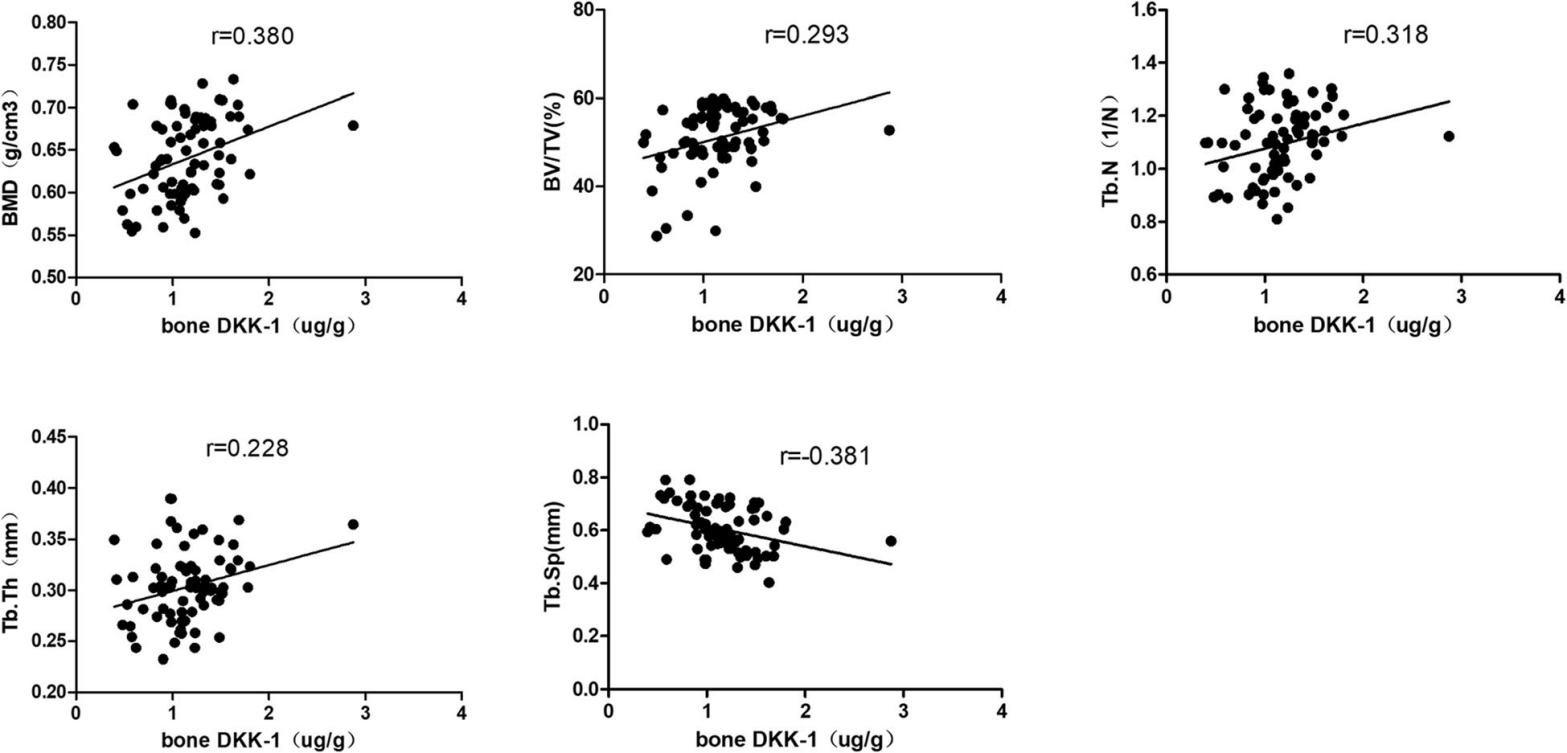
The correlationship of bone Dkk-1 and BMD, bone microarchitecture

Micro-CT was performed on the trabecular bone from the femoral neck, and a representative three-dimensional reconstruction image is shown in Fig. 3. Bone sclerostin was positively correlated with BMD (*r* = 0.355, *P =* 0.002), BV/TV (*r* = 0.401, *P =* 0.000), Tb.N (*r* = 0.329, *P* = 0.004), Mac (*r* = 0.355, *P* = 0.002), and Cos (*r* = 0.293, *P =* 0.01) and negatively correlated with Tb.Sp (*r* = − 0.503, *P =* 0.000) (Fig. 4). Bone Dkk-1 was positively correlated with BMD (*r* = 0.293, *P* = 0.001), BV/TV(*r* = 0.293, *P* = 0.01), Tb.Th (*r* = 0.228, *P =* 0.048), and Tb.N (*r* = 0.318, *P =* 0.005), Mac (*r* = 0.352, *P =* 0.002), Cos (*r* = 0.315, *P =* 0.006), and Yom (*r* = 0.266, *P =* 0.02) and negatively correlated with Tb.Sp (*r* = -0.38, *P* = 0.01) (Fig. 5).

### Correlation between bone sclerostin and Dkk-1 with bone strength

As shown in Table 3, the levels of bone sclerostin and Dkk-1 were positively correlated with the bone strength markers, including Mac, Cos, and Yom.

**Table 3:** Correlation of bone sclerostin and Dkk-1 between BMD, bone microarchitecture and bone strength

	Sclerostin (ug/g)		Dkk-1(ug/g)	
	r	*P*	r	*P*
Mac(N)	0.355	0.05	0.352	0.05
Cos (Mpa)	0.293	0.05	0.315	0.05
Yom (Mpa)	0.194	0.05	0.266	0.05

## Discussion

In this study, the levels of sclerostin and Dkk-1 in the trabecular bone were measured and were found to be positively correlated with BMD, bone microarchitecture, and bone strength. To our knowledge, this study is the first to report the bone protein levels of sclerostin and Dkk-1 in Chinese postmenopausal women and to show the association between protein levels of sclerostin, Dkk-1, and bone microarchitecture and bone strength.

Sclerostin and Dkk-1 are secreted primarily by osteocytes and inhibit bone formation by blocking the osteoblast Wnt/β-catenin signaling pathway [4]. Downregulation of sclerostin and Dkk-1 is associated with significantly increased bone formation. Moreover, it has been demonstrated in both animal models and clinical experiments that the administration of sclerostin antibody is markedly effective in the prevention of osteoporosis after the decrease in estrogen level [22, 34, 35]. In addition, estrogen has been shown to decrease sclerostin expression [13, 14, 18]. Therefore, based on the results of several studies, it is reasonable to expect that sclerostin and Dkk-1 would be inversely correlated with BMD in postmenopausal women. Although a previous study supported this notion that as blood sclerostin and Dkk-1 levels increased, BMD decreased in postmenopausal women, there is more evidence indicating that BMD is positively associated with sclerostin and Dkk-1 [23–29, 36, 37]. Most of these studies analyzed data on serum sclerostin, Dkk-1, and BMD measured by DXA. Considering serum sclerostin and Dkk-1 are also secreted by other cells and tissues, which might not reflect the levels of sclerostin and Dkk-1 in the bone, we measured the protein levels of sclerostin and Dkk-1 in the trabecular bone directly and assessed the local BMD by micro-CT. Our results demonstrated that the serum levels of sclerostin and Dkk-1 could reflect the bone levels of sclerostin and Dkk-1. Consistent with the results from the serum data, we found that the bone protein levels of sclerostin and Dkk-1 were positively associated with BMD. Moreover, these results were also supported by the mRNA levels of sclerostin and Dkk-1 reported by Jemtland et al. [38]. More importantly, the only study that reported the bone protein levels of sclerostin and Dkk-1, published recently, also demonstrated a positive relationship between bone sclerostin and Dkk-1 and BMD [39]. Compared with this study, one advantage of our study was that the specimens were kept in storage for a relatively short time, which could prevent the degradation of the protein levels of sclerostin and Dkk-1. Another paradoxical association discovered in our study was that protein levels of sclerostin and Dkk-1 were negatively associated with age; however, another study reported that serum sclerotin increased with age in men but not in women. We believe that the natural life of osteocytes after estrogen withdrawal and aging led to the paradoxical results mentioned above. Osteocytes constitute more than 90–95% of bone cells in the adult skeleton and are extremely long-lived cells that survive for up to decades in the bone matrix [40]. The life span of osteocytes is most likely determined by the rates of bone turnover, the process by which osteoclasts resorb the bone and osteoblasts replace the resorbed bone [3]. In postmenopausal women, as estrogen declines, bone turnover accelerates, which leads to the apoptosis of osteocytes [3]. It has been shown in bone samples from aged patients with hip fracture that the number of osteocytes and osteocyte activity decreased, whereas osteocyte apoptosis increased [41]. Therefore, a reasonable explanation for the positive correlation of bone sclerostin and Dkk-1 levels with BMD may be that these two proteins are mainly produced by live osteocytes and the number of live osteocytes decrease because of increased apoptosis. Consequently, sclerostin and Dkk-1 levels would reflect the number of osteocytes. From another perspective, when women with PO received teriparatide or denosumab treatment, BMD and sclerostin increased, which was accompanied by reduced bone turnover and increased osteocyte number [42, 43]. Conversely, the number of live osteocytes decreased significantly with age, which might have resulted in the reduced levels of bone sclerostin and Dkk-1.

In addition to BMD, we also evaluated bone microarchitecture and bone strength. Higher levels of sclerostin and Dkk-1 might predict better bone microarchitecture and bone strength. This finding was in accordance with that of Szulc et al., who reported that bone microarchitectural parameters are positively correlated with sclerostin in men [28]. Osteocyte number and activity play a crucial role in determining bone strength and microarchitecture. Ablation of osteocytes rapidly results in decreased bone strength and osteoporosis [44–46]. Hence, as sclerostin and Dkk-1 are mainly secreted by osteocytes, they could reflect the number and activity of osteocytes. Therefore, the bone levels of sclerostin and Dkk-1 could also reflect bone microarchitecture and strength.

However, this study has several limitations. First, no bone specimens were obtained from healthy women as a control group. Second, the number of patients enrolled in the study was relatively low. This might be the reason why we did not find any association between bone sclerosin and Dkk-1 and P1NP and β-CTX, whereas other studies, including a total of 100 patients, reported that serum sclerostin was negatively correlated with β-CTX and P1NP [27, 29]. Third, each patient lacked estrogen dectection, although we confirmed that every patient was postmenopausal according to the medical history; fourth, the patients enrolled in the study were all Chinese women, and our observations originating from bone specimens were consistent with the serum results that were also obtained from Chinese patients; therefore, the results might differ among different races [26, 29].

## Conclusion

Based on the findings of previous studies and our research, bone sclerostin and Dkk-1 were positively correlated with BMD in Chinese women with PO, and higher levels of bone sclerostin and Dkk-1 might predict better BMD, bone microarchitecture, and bone strength. The potential molecular mechanisms still require further study.

## Data Availability

The datasets generated and/or analyzed during the current study are not publicly available but are available from the corresponding author upon request.
